# Target trial emulation studies in oncology: a comprehensive analysis

**DOI:** 10.1093/oncolo/oyag258

**Published:** 2026-07-07

**Authors:** Chenfeng Tan, Zelei Dai, Renjie Zhao, Kefan Li, Bingran Zhang, Ying Wang, Yonggang Zhang, Lei Liu, Nian Li

**Affiliations:** Division of Head and Neck Tumor Multimodality Treatment, Cancer Center, West China Hospital, Sichuan University, Chengdu, 610041, China; Division of Head and Neck Tumor Multimodality Treatment, Cancer Center, West China Hospital, Sichuan University, Chengdu, 610041, China; West China School of Medicine, Sichuan University, Chengdu, 610041, China; West China School of Medicine, Sichuan University, Chengdu, 610041, China; Department of Medical Administration, West China Hospital, Sichuan University, Chengdu, 610041, China; Department of Medical Administration, West China Hospital, Sichuan University, Chengdu, 610041, China; Department of Evidence Based Medicine and Clinical Epidemiology, West China Hospital, Sichuan University, Chengdu, 610041, China; Division of Head and Neck Tumor Multimodality Treatment, Cancer Center, West China Hospital, Sichuan University, Chengdu, 610041, China; Department of Medical Administration, West China Hospital, Sichuan University, Chengdu, 610041, China

**Keywords:** target trial emulation, oncology, real-world evidence

## Abstract

**Background:**

Target trial emulation (TTE) has emerged as an important framework for generating comparative effectiveness real-world evidence. However, the methodological characteristics and clinical utility of this framework in oncology remain insufficiently characterized. This study systematically evaluated the characteristics of oncological emulation studies.

**Materials and methods:**

We systematically searched PubMed, Embase, and Web of Science Core Collection databases from inception to December 22, 2023, to identify oncology-focused TTE studies. Study characteristics, TTE components, and consistency with corresponding randomized controlled trials (RCTs) were analyzed. Categorical variables were summarized as frequencies and percentages and compared using Chi-square tests in SPSS (version 27.0), with *P*-values <0 .05 considered statistically significant.

**Results:**

A total of 54 TTEs were included. Most were conducted in the United States (22, 40.7%), and publications peaked in 2023 (17, 31.5%). Colorectal cancer (13, 24.1%) was the most frequently investigated cancer type. Studies predominantly used data from national databases (18, 33.3%). While all studies prospectively specified the target trial protocol and emulation procedures, only 1 (1.9%) study was prospectively registered, and most emulation studies (48, 88.9%) did not reference a reporting guideline. Among the 10 RCT-TTE pairs available for comparison, 6 (60.0%) pairs achieved agreement in both statistical significance and effect estimates.

**Conclusion:**

This study provides a comprehensive analysis of TTE studies in oncology, highlighting their value for generating higher-quality real-world evidence. Future efforts should prioritize prospective protocol registration and standardize reporting to enhance the credibility and reproducibility of emulation studies.

Implications for Practice Target trial emulation (TTE) is a promising methodological framework for generating higher-quality real-world evidence in oncology, with substantial potential to inform clinical decision-making. Future TTE studies should prioritize prospective protocol registration and adhere to a well-designed reporting guideline to further enhance the transparency, credibility, and reproducibility of oncological research.

## Introduction

Target trial emulation (TTE), proposed by Hernán and Robins in 2016, is a framework for designing and analyzing observational studies to evaluate the comparative effectiveness of interventions by emulating a hypothetical randomized controlled trial (RCT), the target trial, that would ideally answer the clinical question of interest.^1–3^ This framework is implemented in two steps: first, specifying the protocol for the target trial, including eligibility criteria, treatment strategies, treatment assignment, follow-up, primary outcome, causal contrasts, and analysis plan, and second, emulating these components as closely as possible using observational data. By aligning observational study design with RCT principles, TTE can help reduce common biases in observational research and generate real-world evidence with greater clinical interpretability.^4^^,^^5^

The need for reliable real-world evidence is particularly pressing in oncology, where many important comparative effectiveness questions cannot be fully addressed by RCTs due to ethical, logistical, financial, or feasibility constraints.^6^ In addition, oncology is characterized by complex, multi-line treatment pathways influenced by rapid disease progression and treatment-related toxicities, all of which may introduce time-varying confounding in conventional observational analyses.^7^^,^^8^ In this context, the target trial framework provides a structured approach for addressing questions that remain unresolved by randomized trials or by conventional observational studies with conflicting findings.

For oncologists, emulation studies may help bridge the evidence gap between RCT findings and routine clinical practice by generating real-world evidence in patient populations that are often underrepresented in trials, such as older patients and those with comorbidities.^6^ They may also clarify treatment comparisons when direct randomized evidence is unavailable or difficult to obtain, thereby supporting clinical decision-making.^9^ In addition, TTEs may inform the design of future RCTs by refining eligibility criteria, identifying appropriate comparators, and prioritizing treatment strategies for prospective evaluation. Beyond clinical practice, well-designed emulation studies may also support evidence generation for pharmaceutical companies, particularly for innovative or high-cost therapies supported mainly by single-arm trials, by constructing external control arms from observational data, contextualizing treatment effects, and supporting market-access discussions.^10^ For regulatory stakeholders, TTEs may further support post-approval evidence generation by providing causal estimates at relatively low cost, thereby helping evaluate real-world effectiveness, safety, and generalizability in broader patient populations.^11–13^

Despite the growing number of oncological emulation studies, their overall characteristics and application patterns have not been systematically analyzed. More importantly, it remains unclear whether, and to what extent, findings from oncological emulations align with results from corresponding RCTs. To fill these gaps, we comprehensively analyzed published oncological TTEs with the following objectives: (a) to systematically characterize their design and application pattern, (b) to evaluate core target trial components, and (c) to assess consistency between emulation outcomes and corresponding RCT results. By providing a comprehensive overview of current TTEs in oncology and addressing practical implementation challenges, we aim to offer clinical oncologists a valuable reference for interpreting and applying these findings to optimize clinical practice and decision-making.

## Methods

### Literature search

We searched PubMed, Embase, and Web of Science Core Collection databases from their inception until December 22, 2023. These databases were selected for their comprehensive coverage of published studies relevant to our research. The search strategy combined terms representing the concepts of “target trial emulation” OR “hypothetical trials” and “neoplasms” OR “tumor” OR “cancer.” The complete search strategy is available in Supplementary Material 1. Additionally, manual searches were conducted to ensure a comprehensive search.

### Inclusion and exclusion criteria

The inclusion criteria were as follows: (a) retrospective observational studies that applied the target trial framework to emulate a hypothetical target trial, (b) studies that enrolled patients diagnosed with malignancies, evaluated interventions/exposures directly related to oncology, or assessed outcomes related to cancer development, and (c) studies written in English. The exclusion criteria included: (a) non-original research articles, including meeting abstracts, study protocols, methodological papers (methodological papers that aimed to illustrate the TTE framework, without addressing a specific, substantive clinical research question in oncology were excluded), editorials, commentaries, reviews, or theses, (b) articles unavailable in full-text.

### Study screening and data collection

We used EndNote 9.0 software to manage the search records. Two independent reviewers (TCF and DZL) screened titles, abstracts, and full-texts of all studies. Studies meeting the eligibility criteria were included. Any disagreements were resolved by discussion.

Using a predesigned Excel form and guided by the target trial framework proposed by Hernán and Robins,^1^ two reviewers independently extracted data. The process involved identifying and recording the components of the target trial protocol for each study. Any discrepancies were resolved by discussion. The following data were extracted and categorized into three domains: (a) Study characteristics, including publication year, country, data sources, sample size, conflicts of interest, funding, ethics approval, prospective study registration, reporting guidelines, and reasons why a TTE was conducted. (b) Target trial components, including eligibility criteria, treatment strategies, assignment procedures, follow-up periods, primary outcomes, causal contrasts, and analysis plans. (c) Methodological details, including the specification of the target trial protocol and emulation procedures (including their reporting format), sensitivity analyses, and reporting of potential unmeasured confounders.

### Selection of corresponding RCTs and data extraction

For each included study, the two independent reviewers clarified its core research question and summarized the population, intervention, comparison, outcome, and study design (PICOS). We searched PubMed, Web of Science Core Collection, and ClinicalTrials.gov for published RCTs that addressed the same questions.

The RCT inclusion criteria were as follows: (a) study design was an RCT, (b) PIC elements were consistent with the corresponding emulation studies (minor variations were allowed if they were unlikely to alter the question), (c) the study reported outcomes as defined in the corresponding TTE, and (d) the study was cited or identified as the emulated target trial in the TTE study. RCTs were excluded if the results of outcomes could not be extracted or transformed for a comparable effect estimate in emulation studies. Any disagreements were resolved by discussion. The two reviewers then independently extracted data. For the primary outcome, both adjusted and unadjusted hazard ratios (HRs) with their 95% confidence intervals (CIs) were extracted from each TTE study and its corresponding RCT.

### Statistical analysis

We assessed the following outcomes: (a) characteristics of published oncological TTEs, (b) core TTE components, and (c) consistency between TTE and corresponding RCT results.

Consistency was quantified using: (a) statistical significance agreement, defined as both the TTE and corresponding RCT being statistically significant in the same direction or both being statistically nonsignificant, (b) estimate agreement, defined as the TTE adjusted point estimate within the 95% CIs of the RCT result, and (c) standardized difference between the TTE and RCT effect estimates. The methodology adapted from those described by Wang et al.^12^ Categorical variables were summarized as frequency counts and percentages and were compared using Chi-square tests. All analyses were performed using SPSS software (version 27.0), with *P* value <0 .05 considered statistically significant.

## Results

### Study characteristics

The literature search identified a total of 597 studies. After removing duplicates and screening titles, abstracts, and full-texts, 42 studies were identified. Additionally, 12 studies meeting the eligibility criteria were manually included, resulting in a total of 54 included studies.^4^^,^^9^^,^^10^^,^^14–64^ The study flowchart is presented in Figure 1.

**Figure 1.**
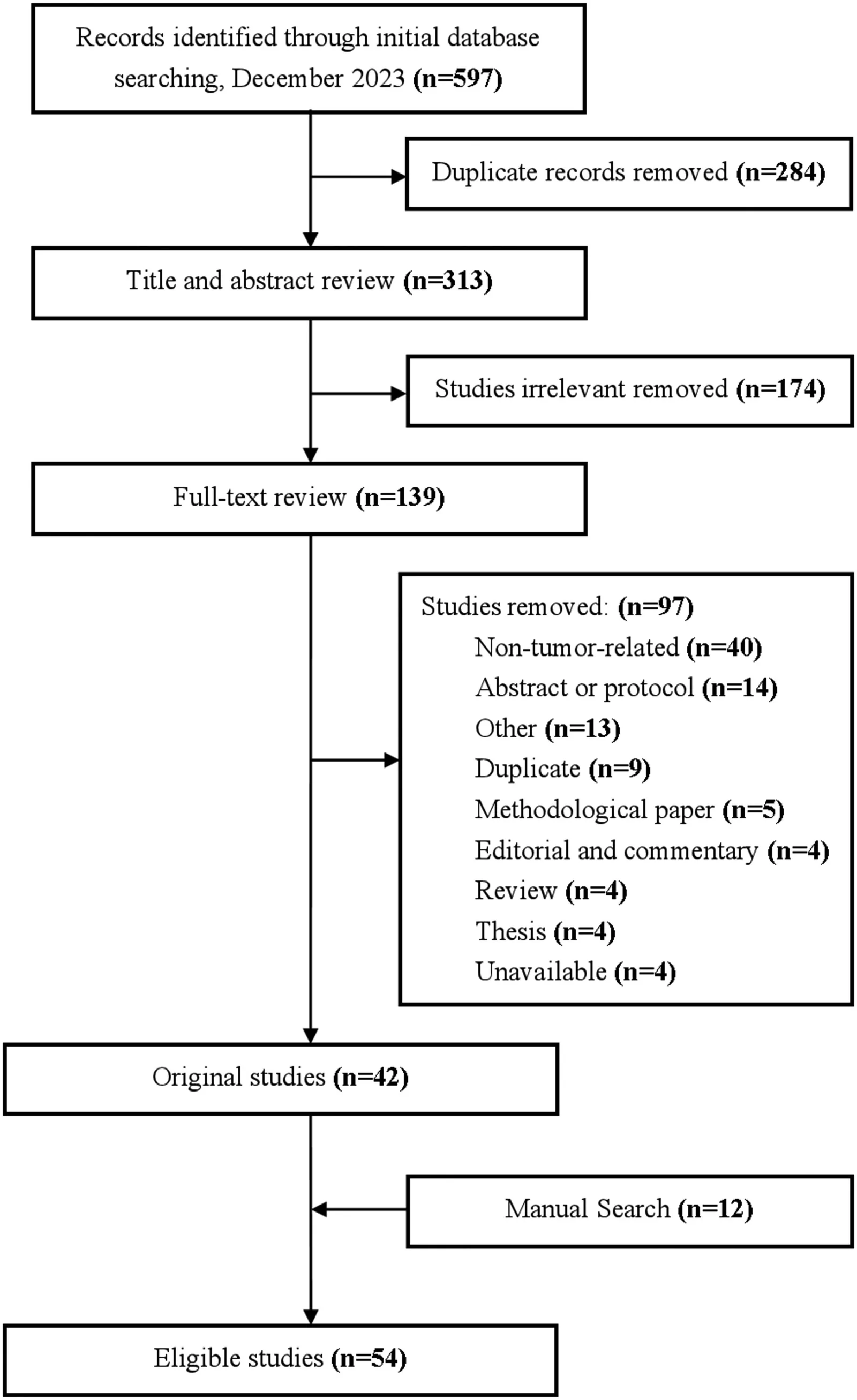
Study flow chart.

Among the included studies, most were conducted in the United States (22, 40.7%), and publications peaked in 2023 (17, 31.5%; Figure 2). Colorectal cancer (13, 24.1%) was the most frequently investigated cancer type. Regarding the study focus, 44 (81.5%) studies focused on treatment, 5 (9.3%) on screening, 2 (3.7%) on prevention, and 1 (1.9%) on medical facilities. In terms of study objective, 45 (83.3%) studies aimed to generate original evidence in the absence of RCTs, whereas only 9 (16.7%) sought to cross-validate or extend the findings of existing RCTs. The included studies predominantly used large-scale observational data sources, with national databases (18, 33.3%), Medicare databases (13, 24.1%), registry studies (10, 18.5%), and electronic health records (EHRs; 9, 16.7%). All studies prospectively specified the target trial protocol and clearly outlined the emulation procedures. However, only 1 (1.9%) study was prospectively registered, and most included studies (48, 88.9%) did not reference a reporting guideline. The detailed characteristics are shown in Table 1 and Supplementary Figure S1.

**Figure 2.**
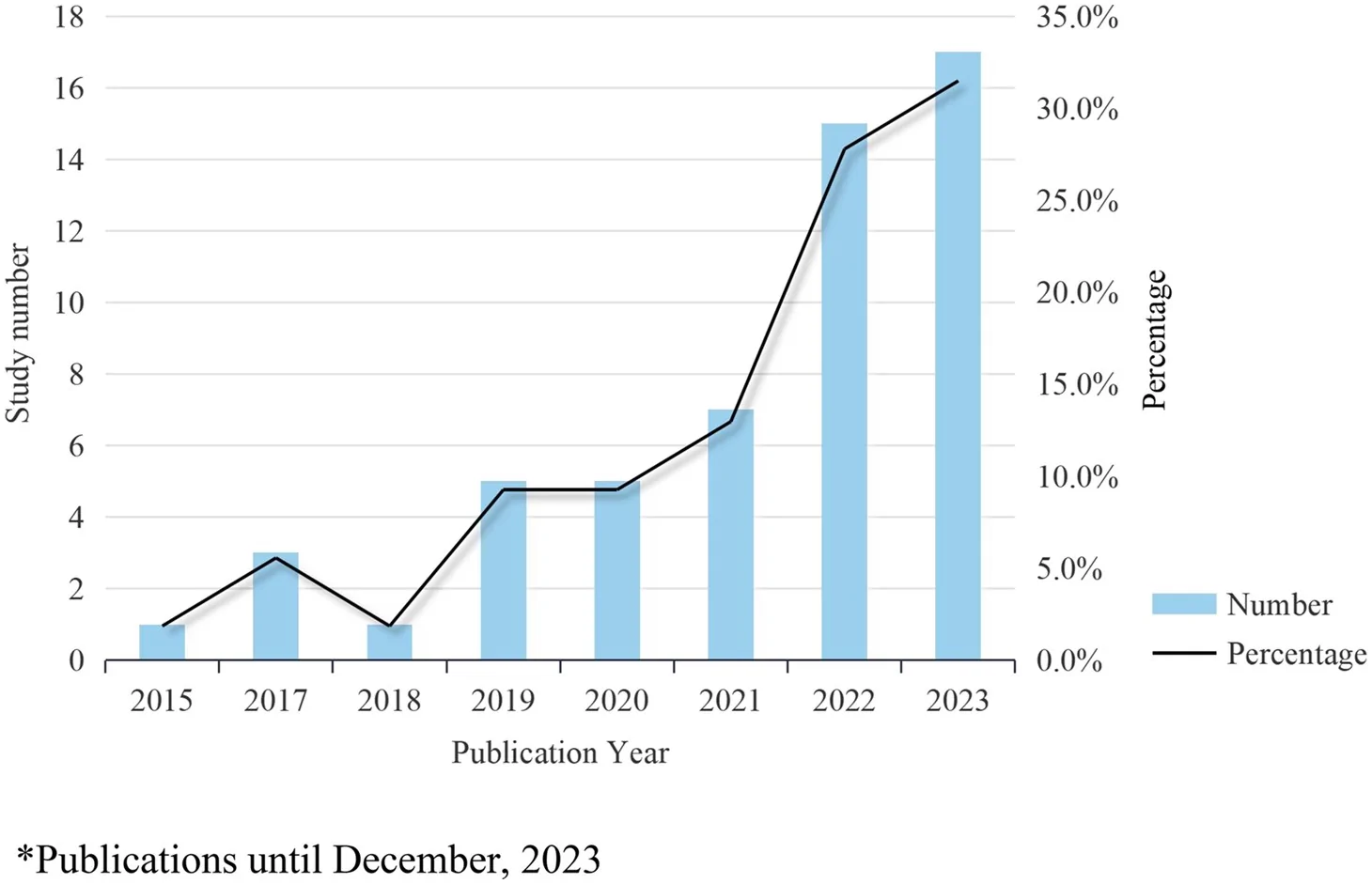
Trend of publication years for the 54 included studies from 2015 to 2023.

**Table 1:** Characteristics of the 54 included studies, 2015-2023.

Characteristics		Number of studies (%)
**Country**		
	United States	22 (40.7)
	Multiple	7 (13.0)
	South Korea	4 (7.4)
	Sweden	4 (7.4)
	Denmark	3 (5.6)
	France	3 (5.6)
	Germany	3 (5.6)
	United Kingdom	3 (5.6)
	Canada	2 (3.7)
	China	1 (1.9)
	Netherlands	1 (1.9)
	Thailand	1 (1.9)
**Cancer type**		
	Colorectal cancer	13 (24.1)
	Prostate cancer	9 (16.7)
	Hepatocellular carcinoma	8 (14.8)
	Pancreatic cancer	5 (9.3)
	Bladder cancer	4 (7.4)
	Multiple	4 (7.4)
	Breast cancer	3 (5.6)
	Esophageal cancer	1 (1.9)
	Intrahepatic cholangiocarcinoma	1 (1.9)
	Lung cancer	1 (1.9)
	Oesophagogastric adenocarcinoma	1 (1.9)
	Ovarian cancer	1 (1.9)
	Renal cell carcinoma	1 (1.9)
	Relapsed/refractory multiple myeloma	1 (1.9)
	Spinal metastasis	1 (1.9)
**Data sources**		
	National databases	18 (33.3)
	Medicare databases	13 (24.1)
	Registry study	10 (18.5)
	EHRs	9 (16.7)
	Clinical trials	3 (5.6)
	Multiple data sources^a^	1 (1.9)
**Conflicts of interests**		
	None^b^	32 (59.3)
	Multiple relationships^c^	12 (22.2)
	Employment relationship	3 (5.6)
	Research support	3 (5.6)
	Consulting relationship	2 (3.7)
	Not mentioned	1 (1.9)
	Other^d^	1 (1.9)
**Funding sources**		
	Other institutional and foundation funding^e^	18 (33.3)
	Not mentioned	12 (22.2)
	Government funding	10 (18.5)
	Multiple funding sources^f^	9 (16.7)
	Corporate sponsorship	3 (5.6)
	None	2 (3.7)
**Ethics approval**		
	Ethics approved	34 (63.0)
	Exempt from ethics approval	16 (29.6)
	Not mentioned	4 (7.4)
**Study registration status**		
	Yes	1 (1.9)
	No	53 (98.1)
**Reporting guideline**		
	Not mentioned	48 (88.9)
	STROBE	2 (3.7)
	STROBE and ISPOR	2 (3.7)
	ISPOR	1 (1.9)
	RECORD	1 (1.9)
**Specification of the emulation procedures**		
	Yes	54 (100.0)
	No	0 (0)
**Specification of the target trial protocol**		
	Yes	54 (100.0)
	No	0 (0)
**Reporting format of the protocol**		
	Table	34 (63.0)
	Text	20 (37.0)
**Sensitivity analyses**		
	Yes	45 (83.3)
	No	9 (16.7)
**Reporting of potential unmeasured confounders**		
	Yes, without *E*-value	50 (92.6)
	Yes, with *E*-value	4 (7.4)

Abbreviations: EHR, electronic health record; ISPOR, International Society for Pharmacoeconomics and Outcomes Research; RCT, randomized controlled trial; RECORD, reporting of studies conducted using routinely collected data; STROBE, strengthening the reporting of observational studies in epidemiology; TTE, target trial emulation.

a Studies using multiple data sources, including EHRs, national databases, health insurance databases, or clinical research data.

b Studies reporting no conflicts of interest.

c Studies reporting multiple types of conflicts of interest, including employment, research support, consulting relationships, or editorial affiliations.

d Studies reporting other conflicts of interest not categorized as employment, research support, consulting relationships, or editorial affiliations.

e Funding from institutional, foundation, or other sources excluding government and corporate sources.

f Funding from multiple sources, including government, corporate, institutional, or foundation sources.

### Components of TTE

Following the target trial framework proposed by Hernán and Robins,^1^ we systematically reviewed the key protocol components across the 54 included studies. Regarding treatment strategies, comparisons were categorized into two types: presence-absence intervention comparisons (34, 63.0%) and intervention modality/detail variations (20, 37.0%). The investigated strategies contained: pharmacology (26, 48.1%), surgery (9, 16.7%), cross-modal therapies (5, 9.3%), screening strategies (5, 9.3%), radiotherapy (4, 7.4%), lifestyle modifications (2, 3.7%), supportive care (2, 3.7%), and medical facility utilization (1, 1.9%). The most frequently assessed primary outcomes were mortality (35, 64.8%) and cancer incidence (13, 24.1%), with fewer studies assessing patient well-being (5, 9.3%) or pathological response (1, 1.9%). Follow-up periods ranged from 30 days to 15 years, depending on the specific research question. Detailed information regarding bias adjustments is presented in Supplementary Table S1.

### Consistency assessment

Among the 54 included studies, nine TTEs corresponded to published RCTs with comparable outcome data. One TTE study emulated two separate RCTs, resulting in a total of 10 RCT-TTE comparison pairs. Among these pairs, 6 (60.0%) achieved agreement in both statistical significance and effect estimates (dual consistency). All 10 (100.0%) pairs achieved estimate agreement, whereas 6 (60.0%) achieved statistical agreement. Detailed information is presented in Table 2.

**Table 2:** The consistency between TTE and corresponding RCT results.

Study	**Effect estimates (95% CIs)**					Agreement metrics		
	Trial name/NCT number	RCT	TTE adjusted	TTE crude	Standardized difference	Statistical significance	Estimate	Standardized- difference
**1^64^**	QUASAR	1.02 (0.70-1.48)	0.95 (0.85-1.04)	1.00 (0.89-1.12)	0.36	SA	EA	SD
**2^64^**	Erlotinib trial	0.96 (0.74-1.24)	1.04 (0.86-1.42)	1.08 (0.93-1.25)	−0.42	SA	EA	SD
**3^43^**	NCT01734798	0.61 (0.33-1.11)	1.11 (0.82-1.51)	1.44 (1.07-1.92)	−1.90	SA	EA	SD
**4^28^**	EORTC 30881	1.02 (0.80-1.29)	1.13 (1.07-1.20)	1.26 (1.20-1.33)	−1.38	N	EA	SD
**5^41^**	IDEA	0.96 (0.85-1.08)	0.96 (0.43-2.14)	NA	0.01	SA	EA	SD
**6^14^**	EORTC 55955	1.01 (0.82-1.25)	1.12 (0.96-1.28)	NA	−0.78	SA	EA	SD
**7^15^**	NCT00002575	0.91 (0.68-1.21)	0.84 (0.79-0.89)	NA	0.68	N	EA	SD
**8^54^**	NCT02491983	1.00 (0.68-1.48)	1.07 (0.86-1.35)	1.24 (1.02-1.51)	−0.30	SA	EA	SD
**9^57^**	NCT00309985	0.72 (0.59-0.89)	0.71 (0.42-1.19)	0.68 (0.45-1.03)	0.09	N	EA	SD
**10^59^**	NCT00028990	0.88 (0.74-1.05)	0.84 (0.77-0.92)	0.89 (0.82-0.98)	0.71	N	EA	SD

Abbreviations: CIs, confidence intervals; EA, estimate agreement, with TTE adjusted point estimates falling within the 95% CIs of the corresponding RCT result; SA, statistical significance agreement, with both the TTE and corresponding RCT being statistically significant in the same direction or both being statistically nonsignificant; N, No; NA, not applicable; NCT, national clinical trial; RCT, randomized controlled trial; SD, standardized-differences agreement, defined by standardized differences |*Z*| < 1.96; TTE, target trial emulation.

## Discussion

### Overview of TTE in oncology

Our study demonstrated that the target trial framework has become an important approach for filling evidence gaps in oncology. Most included studies focused on therapeutic interventions with long-term survival outcomes and used large-scale real-world data. These studies were particularly relevant to clinical, regulatory, and health-technology decision-making when randomized evidence is unavailable or insufficient.

This framework is especially valuable for addressing oncology questions that are difficult to answer in randomized trials, including studies of underrepresented populations, head-to-head comparisons of treatment regimens, complex treatment strategies, long-term outcome assessments, and drug repurposing.^4^^,^^40^^,^^42^^,^^44^^,^^65^^,^^66^ For example, Medicare data have been used to emulate target trials comparing adjuvant fluorouracil versus observation for stage II colorectal cancer and evaluating the addition of erlotinib to gemcitabine for advanced pancreatic cancer in older populations.^64^ TTEs also enable head-to-head comparisons of routine clinical regimens. An example is the comparison between FOLFIRINOX (folinic acid, fluorouracil, irinotecan, and oxaliplatin) and GN (gemcitabine plus nab-paclitaxel) for advanced pancreatic carcinoma, filling an important comparative-effectiveness gap left by separate trials.^9^ Beyond these examples, this method may help evaluate treatment sequences, cycle numbers, dosing, dynamic treatment strategies influenced by disease progression and patient adherence, long-term outcomes, and potential drug repurposing opportunities.^40^^,^^44^^,^^65^^,^^66^

Overall, emulation studies should be regarded as structured complements to RCTs rather than substitutes for them. Their value lies in translating clinically important but otherwise difficult-to-test questions into explicitly specified target trial protocols that can be implemented using observational data.

### Challenges of TTE in oncology

Despite its utility, current applications of TTE in oncology face several challenges. First, data relevance and reliability determine whether the emulation can appropriately answer the intended clinical question.^67^^,^^68^ Oncological research requires not only demographic information but also a broad range of clinical variables, such as medical history, tumor characteristics, and comorbidities. However, national databases, the primary data sources for these studies, often suffer from missing clinical variables and incomplete treatment records, introducing unmeasured biases.^69^ Future emulation studies should prioritize data standardization and integrate multi-source linked data to enhance data quality.

Second, clearly defining “time zero” is essential in target trial emulation.^1^ Failure to synchronize eligibility, treatment assignment, and follow-up can introduce immortal time bias.^8^ Researchers should establish standardized, clinically meaningful “time zero” events (e.g., date of diagnosis, first treatment, or enrollment) and conduct sensitivity analyses to assess the robustness of the results.

Finally, the transparency and reproducibility of emulation studies remain insufficient. Our study showed that prospective registration and use of reporting guidelines were uncommon in oncological TTEs. Pre-specifying the target trial protocol before outcome analysis can enable independent appraisal of analytic decisions, reduce publication bias, and improve study credibility. Moreover, standardized reporting guidelines are still lacking despite the growing application of TTE.^70^ Transparent reporting of the study protocol and its emulation process is crucial to enhance the interpretability and utility of findings.^70–73^ Recently, the Transparent Reporting of Studies Emulating a Target Trial (TARGET) reporting guideline represents a promising tool to standardize TTE reporting.^74^

### Guidance for clinical practice

Oncologists should interpret TTEs as well-designed observational studies, not as true RCTs, since these studies can never completely replicate blinding and randomization. Specifically, when assessing such studies, clinicians should rigorously evaluate the data source quality, the definition of “time zero,” analysis plans, and reporting transparency. Findings from emulations with small sample sizes, low emulation fidelity, or extrapolated beyond the target population should not be applied to clinical practice.^1^ Only through such rigorous appraisal can emulation studies effectively fulfill their role as a key complement to RCTs, successfully integrating real-world evidence into cancer prevention, screening, and treatment.

### Limitations

This study provided a comprehensive overview of current oncological emulation studies; however, several limitations should be acknowledged. First, our assessment of TTE characteristics and methodological quality was based on reported information. Incomplete or suboptimal reporting may have led to misclassification of certain design features or analytic approaches. Second, the number of TTEs with corresponding published RCTs was limited, which constrained the statistical power of the consistency assessments.

## Conclusion

In conclusion, the present study provides a comprehensive analysis of the application of the target trial framework within the oncology literature. Our findings demonstrate that the TTE framework should be considered a promising methodology for generating high-quality real-world evidence, particularly when RCTs are not feasible. Future efforts should prioritize the prospective registration of emulation studies to further bolster the credibility and reproducibility of real-world research.

## Supplementary Material

oyag258_Supplementary_Data

## Data Availability

The data is available upon reasonable request to the corresponding author.

## References

[1] Hernan MA , Robins JM. Using big data to emulate a target trial when a randomized trial is not available. Am J Epidemiol. 2016;183:758-764. 10.1093/aje/kwv254 PMC4832051 26994063

[2] Hernán MA. Methods of public health research - strengthening causal inference from observational data. N Engl J Med. 2021;385:1345-1348. 10.1056/NEJMp2113319 34596980

[3] Zuo H , Yu L, Campbell SM, Yamamoto SS, Yuan Y. The implementation of target trial emulation for causal inference: a scoping review. J Clin Epidemiol. 2023;162:29-37. 10.1016/j.jclinepi.2023.08.003 37562726

[4] Maringe C , Majano SB, Exarchakou A, et al Reflection on modern methods: trial emulation in the presence of immortal-time bias. Assessing the benefit of major surgery for elderly lung cancer patients using observational data. Int J Epidemiol. 2020;49:1719-1729. 10.1093/ije/dyaa057 PMC7823243 32386426

[5] Kennedy-Martin T , Curtis S, Faries D, Robinson S, Johnston J. A literature review on the representativeness of randomized controlled trial samples and implications for the external validity of trial results. Trials. 2015;16:495. 10.1186/s13063-015-1023-4 PMC4632358 26530985

[6] Tan YY , Papez V, Chang WH, Mueller SH, Denaxas S, Lai AG. Comparing clinical trial population representativeness to real-world populations: an external validity analysis encompassing 43 895 trials and 5 685 738 individuals across 989 unique drugs and 286 conditions in England. Lancet Healthy Longev. 2022;3:e674-e689. 10.1016/S2666-7568(22)00186-6 36150402

[7] Ahmad TA , Gopal DP, Chelala C, Dayem Ullah AZ, Taylor SJ. Multimorbidity in people living with and beyond cancer: a scoping review. Am J Cancer Res. 2023;13:4346-4365. PMC10560952 37818046

[8] Hernan MA , Sauer BC, Hernandez-Diaz S, Platt R, Shrier I. Specifying a target trial prevents immortal time bias and other self-inflicted injuries in observational analyses. J Clin Epidemiol. 2016;79:70-75. 10.1016/j.jclinepi.2016.04.014 PMC5124536 27237061

[9] Boyne DJ , Brenner DR, Gupta A, et al Head-to-head comparison of FOLFIRINOX versus gemcitabine plus nab-paclitaxel in advanced pancreatic cancer: a target trial emulation using real-world data. Ann Epidemiol. 2023;78:28-34. 10.1016/j.annepidem.2022.12.005 36563766

[10] Hao Y , Hsu WC, Parzynski CS, et al Effectiveness of tisagenlecleucel versus real-world standard of care in relapsed/refractory follicular lymphoma. J Comp Eff Res. 2023;12:e220173. 10.57264/cer-2022-0173 PMC10508300 37345672

[11] Makary MA , Prasad V. Priorities for a new FDA. JAMA. 2025;334:565-566. 10.1001/jama.2025.10116 40493446

[12] Wang SV , Schneeweiss S, Franklin JM, et al; RCT-DUPLICATE Initiative. Emulation of randomized clinical trials with nonrandomized database analyses: results of 32 clinical trials. JAMA. 2023;329:1376-1385. 10.1001/jama.2023.4221 PMC10130954 37097356

[13] Barosa M , Prasad V. The cost of emulated target trials: is real-world data cheaper than randomized studies? Eur J Clin Invest. 2025;55:e70035. 10.1111/eci.70035 40130313

[14] Kuehne F , Arvandi M, Hess LM, et al Causal analyses with target trial emulation for real-world evidence removed large self-inflicted biases: systematic bias assessment of ovarian cancer treatment effectiveness. J Clin Epidemiol. 2022;152:269-280. 10.1016/j.jclinepi.2022.10.005 36252741

[15] Hou J , Zhao R, Cai T, et al Temporal trends in clinical evidence of 5-year survival within electronic health records among patients with early-stage colon cancer managed with laparoscopy-assisted colectomy vs open colectomy. JAMA Netw Open. 2022;5:E2218371. 10.1001/jamanetworkopen.2022.18371 PMC9227003 35737384

[16] Dickerman BA , Garcia-Albeniz X, Logan RW, Denaxas S, Hernan MA. Avoidable flaws in observational analyses: an application to statins and cancer. Nat Med. 2019;25:1601-1606. 10.1038/s41591-019-0597-x PMC7076561 31591592

[17] Boernhorst C , Reinders T, Rathmann W, et al Avoiding time-related biases: a feasibility study on antidiabetic drugs and pancreatic cancer applying the parametric g-formula to a large German healthcare database. Clin Epidemiol. 2021;13:1027-1038. 10.2147/CLEP.S328342 PMC8560066 34737647

[18] Jang HY , Lee HK, Kim CJ, Yoon SS, Kim IW, Oh JM. Carfilzomib’s real-world safety outcomes in Korea: target trial emulation study using electronic health records. Int J Environ Res Public Health. 2022;19:13560. 10.3390/ijerph192013560 PMC9603615 36294140

[19] Edeline J , Bridgewater J, Campillo-Gimenez B, et al Chemotherapy with or without selective internal radiation therapy for intrahepatic cholangiocarcinoma: data from clinical trials. Hepatology. 2024;79:96-106. 10.1097/HEP.0000000000000544 37505216

[20] Madenci AL , Wanis KN, Cooper Z, et al Comparison of mortality risk with different surgeon and hospital operative volumes among individuals undergoing pancreatectomy by emulating target trials in US medicare beneficiaries. JAMA Netw Open. 2022;5:e221766. 10.1001/jamanetworkopen.2022.1766 PMC8914572 35267034

[21] García-Albéniz X , Hernán MA, Logan RW, Price M, Armstrong K, Hsu J. Continuation of annual screening mammography and breast cancer mortality in women older than 70 years. Ann Intern Med. 2020;172:381-389. 10.7326/M18-1199 32092767

[22] Kirkegård J , Ladekarl M, Johannsen IR, Mortensen F. Effect of adjuvant chemotherapy after pancreatectomy in patients with node-negative pancreatic cancer: target trial emulation. Br J Surg. 2024;111:znad398. 10.1093/bjs/znad398 38006324

[23] Lazzati A , Epaud S, Ortala M, Katsahian S, Lanoy E. Effect of bariatric surgery on cancer risk: results from an emulated target trial using population-based data. Br J Surg. 2022;109:433-438. 10.1093/bjs/znac003 35136932

[24] Park Y , Kang D, Sinn DH, et al Effect of lifestyle modification on hepatocellular carcinoma incidence and mortality among patients with chronic hepatitis B. World J Gastroenterol. 2023;29:3843-3854. 10.3748/wjg.v29.i24.3843 PMC10324530 37426323

[25] García-Albéniz X , Hsu J, Bretthauer M, Hernán MA. Effectiveness of screening colonoscopy to prevent colorectal cancer among medicare beneficiaries aged 70 to 79 years: a prospective observational study. Ann Intern Med. 2017;166:18-26. 10.7326/M16-0758 PMC5417337 27669524

[26] Kraglund F , Christensen DH, Eiset AH, Villadsen GE, West J, Jepsen P. Effects of statins and aspirin on HCC risk in alcohol-related cirrhosis: nationwide emulated trials. Hepatol Commun. 2023;7:e0013. 10.1097/HC9.0000000000000013 PMC9827970 36633465

[27] Dickerman BA , Garcia-Albeniz X, Logan RW, Denaxas S, Hernan MA. Emulating a target trial in case-control designs: an application to statins and colorectal cancer. Int J Epidemiol. 2020;49:1637-1646. 10.1093/ije/dyaa144 PMC7746409 32989456

[28] Bacic J , Liu T, Thompson RH, et al Emulating target clinical trials of radical nephrectomy with or without lymph node dissection for renal cell carcinoma. Urology. 2020;140:98-106. 10.1016/j.urology.2020.01.039 PMC7255934 32142726

[29] Buranupakorn T , Thangsuk P, Patumanond J, Phinyo P. Emulation of a target trial to evaluate the causal effect of palliative care consultation on the survival time of patients with hepatocellular carcinoma. Cancers (Basel). 2021;13:1-15. 10.3390/cancers13050992 PMC7956840 33673534

[30] Smith LH , Garcia-Albeniz X, Chan JM, et al Emulation of a target trial with sustained treatment strategies: an application to prostate cancer using both inverse probability weighting and the g-formula. Eur J Epidemiol. 2022;37:1205-1213. 10.1007/s10654-022-00929-7 36289138

[31] Dickerman BA , Giovannucci E, Pernar CH, Mucci LA, Hernan MA. Guideline-Based physical activity and survival among US men with nonmetastatic prostate cancer. Am J Epidemiol. 2019;188:579-586. 10.1093/aje/kwy261 PMC6395165 30496346

[32] Schaufler C , Kaul S, Fleishman A, et al Immediate radiotherapy versus observation in patients with node-positive prostate cancer after radical prostatectomy. *Prostate Cancer Prostatic Dis*. 2024;27:81-88. 10.1038/s41391-022-00619-1 36434164

[33] Pilleron S , Maringe C, Morris EJA, Leyrat C. Immortal-time bias in older vs younger age groups: a simulation study with application to a population-based cohort of patients with Colon cancer. Br J Cancer. 2023;128:1521-1528. 10.1038/s41416-023-02187-0 PMC10070415 36759725

[34] Kirkegard J , Ladekarl M, Lund A, Mortensen F. Impact on survival of early versus late initiation of adjuvant chemotherapy after pancreatic adenocarcinoma surgery: a target trial emulation. Ann Surg Oncol. 2024;31:1310-1318. 10.1245/s10434-023-14497-x PMC10761389 37914923

[35] Heil TC , Verdaasdonk EGG, Maas HAAM, et al Improved postoperative outcomes after prehabilitation for colorectal cancer surgery in older patients: an emulated target trial. Ann Surg Oncol. 2023;30:244-254. 10.1245/s10434-022-12623-9 PMC9533971 36197561

[36] Yang CT , Yao WY, Yang CY, Peng ZY, Ou HT, Kuo S. Lower risks of cirrhosis and hepatocellular carcinoma with GLP-1RAs in type 2 diabetes: a nationwide cohort study using target trial emulation framework. J Intern Med. 2024;295:357-368. 10.1111/joim.13751 37994187

[37] Russell B , Sherif A, Häggström C, et al Neoadjuvant chemotherapy for muscle invasive bladder cancer: a nationwide investigation on survival. Scand J Urol. 2019;53:206-212. 10.1080/21681805.2019.1624611 31174452

[38] Sanchez A , Roos VH, Navarro M, et al Quality of colonoscopy is associated with adenoma detection and postcolonoscopy colorectal cancer prevention in lynch syndrome. Clin Gastroenterol Hepatol. 2022;20:611-621.e9. 10.1016/j.cgh.2020.11.002 33157315

[39] Stattin P , Westerberg M, Lissbrant IF, et al Real world outcomes in patients with metastatic, castration-resistant prostate cancer treated with radium-223 in routine clinical practice in Sweden. Clin Genitourin Cancer. 2023;21:107.e1-107.e9. 10.1016/j.clgc.2022.09.002 36180341

[40] Reitblat C , Fleishman A, Kaplan IA, et al Radical prostatectomy versus external beam radiation therapy for high-grade, clinically localized prostate cancer: emulation of a target clinical trial. Urol Oncol. 2021;39:785.e1-785.e10. 10.1016/j.urolonc.2021.03.017 33934965

[41] Boyne DJ , Cheung WY, Hilsden RJ, et al Association of a shortened duration of adjuvant chemotherapy with overall survival among individuals with stage III colon cancer. JAMA Netw Open. 2021;4:e213587. 10.1001/jamanetworkopen.2021.3587 PMC8010592 33783516

[42] Corre F , Albouys J, Tran VT, et al Impact of surgery after endoscopically resected high-risk T1 colorectal cancer: results of an emulated target trial. Gastrointest Endosc. 2024;99:408-416.e2. 10.1016/j.gie.2023.09.027 37793506

[43] Ernandez J , Kaul S, Fleishman A, et al Adjuvant chemotherapy plus radiotherapy versus chemotherapy alone for locally advanced bladder cancer after radical cystectomy. Bladder Cancer. 2022;8:405-417. 10.3233/BLC-220031 PMC11181795 38994178

[44] Garcia-Albeniz X , Chan JM, Paciorek A, et al Immediate versus deferred initiation of androgen deprivation therapy in prostate cancer patients with PSA-only relapse. An observational follow-up study. Eur J Cancer. 2015;51:817-824. 10.1016/j.ejca.2015.03.003 PMC4402138 25794605

[45] García-Albéniz X , Hsu J, Hernán MA. The value of explicitly emulating a target trial when using real world evidence: an application to colorectal cancer screening. Eur J Epidemiol. 2017;32:495-500. 10.1007/s10654-017-0287-2 PMC5759953 28748498

[46] Emilsson L , García-Albéniz X, Logan RW, Caniglia EC, Kalager M, Hernán MA. Examining bias in studies of statin treatment and survival in patients with cancer. JAMA Oncol. 2018;4:63-70. 10.1001/jamaoncol.2017.2752 PMC5790310 28822996

[47] Schoenfeld AJ , Losina E, Ferrone ML, et al Ambulatory status after surgical and nonsurgical treatment for spinal metastasis. Cancer. 2019;125:2631-2637. 10.1002/cncr.32140 PMC6625859 30985913

[48] Thomsen FB , Bosco C, Garmo H, et al Anti-androgen monotherapy versus gonadotropin-releasing hormone agonists in men with advanced, non-metastatic prostate cancer: a register-based, observational study. Acta Oncol. 2019;58:110-118. 10.1080/0284186X.2018.1529427 30375907

[49] Pak S , You D, Jeong IG, et al Adjuvant chemotherapy versus observation after radical cystectomy in patients with node-positive bladder cancer. Sci Rep. 2019;9:8305. 10.1038/s41598-019-44504-9 PMC6549178 31165753

[50] Boström P , Hultberg DK, Häggström J, et al Oncological impact of high vascular tie after surgery for rectal cancer: a nationwide cohort study. Ann Surg. 2021;274:e236-e244. 10.1097/SLA.0000000000003663 34397455

[51] Kwee SA , Wong LL, Sato MM, et al Transarterial radioembolization for hepatocellular carcinoma with major vascular invasion: a nationwide propensity score-matched analysis with target trial emulation. J Vasc Interv Radiol. 2021;32:1258-1266.e6. 10.1016/j.jvir.2021.07.001 PMC8410665 34242775

[52] Rahman S , Thomas B, Maynard N, et al Impact of postoperative chemotherapy on survival for oesophagogastric adenocarcinoma after preoperative chemotherapy and surgery. Br J Surg. 2022;109:227-236. 10.1093/bjs/znab427 PMC10364695 34910129

[53] Gaber CE , Shaheen NJ, Edwards JK, et al Trimodality therapy vs definitive chemoradiation in older adults with locally advanced esophageal cancer. JNCI Cancer Spectr. 2022;6:pkac069. 10.1093/jncics/pkac069 PMC9623425 36205723

[54] Merola D , Young J, Schrag D, Lin KJ, Robert N, Schneeweiss S. Oncology drug effectiveness from electronic health record data calibrated against RCT evidence: the PARSIFAL trial emulation. Clin Epidemiol. 2022;14:1135-1144. 10.2147/CLEP.S373291 PMC9563733 36246306

[55] Softness K , Kaul S, Fleishman A, et al Radical cystectomy versus trimodality therapy for muscle-invasive urothelial carcinoma of the bladder. Urol Oncol. 2022;40:272.e1-272.e9. 10.1016/j.urolonc.2021.12.015 35058142

[56] Braitmaier M , Schwarz S, Kollhorst B, Senore C, Didelez V, Haug U. Screening colonoscopy similarly prevented distal and proximal colorectal cancer: a prospective study among 55-69-year-olds. J Clin Epidemiol. 2022;149:118-126. 10.1016/j.jclinepi.2022.05.024 35680106

[57] Ghiani M , Maywald U, Wilke T, Heeg B. Bridging the gap between oncology clinical trials and real-world data: evidence on replicability of efficacy results using German claims data. J Comp Eff Res. 2022;11:513-521. 10.2217/cer-2021-0224 35315280

[58] Lu Y , Gehr AW, Meadows RJ, et al Timing of adjuvant chemotherapy initiation and mortality among Colon cancer patients at a safety-net health system. BMC Cancer. 2022;22:593. 10.1186/s12885-022-09688-w PMC9158363 35641921

[59] Antoine A , Pérol D, Robain M, Delaloge S, Lasset C, Drouet Y. Target trial emulation to assess real-world efficacy in the epidemiological strategy and medical economics metastatic breast cancer cohort. J Natl Cancer Inst. 2023;115:971-980. 10.1093/jnci/djad092 PMC10407701 37220893

[60] Sinn DH , Kang D, Park Y, et al Statin use and the risk of hepatocellular carcinoma among patients with chronic hepatitis B: an emulated target trial using longitudinal nationwide population cohort data. BMC Gastroenterol. 2023;23:366. 10.1186/s12876-023-02996-w PMC10601275 37880589

[61] Emilsson L , Song M, Ludvigsson JF. Target trial emulation of aspirin after diagnosis of colorectal polyps. Eur J Epidemiol. 2023;38:1105-1114. 10.1007/s10654-023-01024-1 PMC10570175 37322135

[62] Kwee SA , Wong LL, Ludema C, et al Target trial emulation: a design tool for cancer clinical trials. JCO Clin Cancer Inform. 2023;7:e2200140. 10.1200/CCI.22.00140 PMC10166475 36608311

[63] Carlsson S , Bock D, Lantz A, et al Salvage radiotherapy after radical prostatectomy: functional outcomes in the LAPPRO trial after 8-year follow-up. Scand J Urol. 2023;58:11-19. 10.2340/sju.v58.7318 37170656

[64] Petito LC , García-Albéniz X, Logan RW, et al Estimates of overall survival in patients with cancer receiving different treatment regimens: emulating hypothetical target trials in the surveillance, epidemiology, and end results (SEER)-medicare linked database. JAMA Netw Open. 2020;3:e200452. 10.1001/jamanetworkopen.2020.0452 PMC7059023 32134464

[65] Tan GSQ , Sloan EK, Lambert P, Kirkpatrick CMJ, Ilomäki J. Drug repurposing using real-world data. Drug Discov Today. 2023;28:103422. 10.1016/j.drudis.2022.103422 36341896

[66] Ozery-Flato M , Goldschmidt Y, Shaham O, Ravid S, Yanover C. Framework for identifying drug repurposing candidates from observational healthcare data. JAMIA Open. 2020;3:536-544. 10.1093/jamiaopen/ooaa048 PMC7886555 33623890

[67] Gomes M , Latimer N, Soares M, et al Target trial emulation for transparent and robust estimation of treatment effects for health technology assessment using real-world data: opportunities and challenges. Pharmacoeconomics. 2022;40:577-586. 10.1007/s40273-022-01141-x 35332434

[68] Girman CJ , Ritchey ME, Lo Re V III. Real-world data: assessing electronic health records and medical claims data to support regulatory decision-making for drug and biological products. Pharmacoepidemiol Drug Saf. 2022;31:717-720. 10.1002/pds.5444 PMC9320939 35471704

[69] Galozy A , Nowaczyk S, Sant’Anna A, Ohlsson M, Lingman M. Pitfalls of medication adherence approximation through EHR and pharmacy records: definitions, data and computation. Int J Med Inform. 2020;136:104092. 10.1016/j.ijmedinf.2020.104092 32062562

[70] Hansford HJ , Cashin AG, Jones MD, et al Reporting of observational studies explicitly aiming to emulate randomized trials: a systematic review. JAMA Netw Open. 2023;6:e2336023. 10.1001/jamanetworkopen.2023.36023 PMC10534275 37755828

[71] Crown W , Dahabreh IJ, Li X, Toh S, Bierer B. Can observational analyses of routinely collected data emulate randomized trials? Design and feasibility of the observational patient evidence for regulatory approval science and understanding disease project. Value Health. 2023;26:176-184. 10.1016/j.jval.2022.07.003 35970705

[72] Pearce N , Vandenbroucke JP. Are target trial emulations the gold standard for observational studies? Epidemiology. 2023;34:614-618. 10.1097/EDE.0000000000001636 37255243

[73] De Stavola BL , Gomes M, Katsoulis M. Transparency and rigor: target trial emulation aims to achieve both. Epidemiology. 2023;34:624-626. 10.1097/EDE.0000000000001638 37255253

[74] Hansford HJ , Cashin AG, Jones MD, et al Development of the TrAnsparent ReportinG of observational studies emulating a target trial (TARGET) guideline. BMJ Open. 2023;13:e074626. 10.1136/bmjopen-2023-074626 PMC10503363 37699620

