# Supplementary material for: Target trial emulation studies in oncology: a comprehensive analysis
Source: Oncologist. 2026 Jul 7;31(11):oyag258. doi: 10.1093/oncolo/oyag258 (PMC13645428; doi:10.1093/oncolo/oyag258)
Supplement: Supplementary file 1 [file oyag258_supplementary_data.zip › Supplementary Table.docx]

**Supplementary Table 1**. TTE Components in the 54 Included Studies, 2015-2023.

**Supplementary Table 1**. TTE Components in the 54 Included Studies, 2015-2023.

| Items |  | No. % |
| --- | --- | --- |
|  |  | Total study  n=54 (100) |
| Grace period- |  |  |
|  | Yes | 27 (50.0) |
|  | No | 27 (50.0) |
| Treatment strategy comparisons |  |  |
|  | Presence-absence intervention comparisons | 34 (63.0) |
|  | Intervention modality/detail variations | 20 (37.0) |
| Treatment strategies |  |  |
|  | Pharmacological | 26 (48.1) |
|  | Surgical | 9 (16.7) |
|  | ^a^Cross-modal | 5 (9.3) |
|  | Screening | 5 (9.3) |
|  | Radiotherapy | 4 (7.4) |
|  | Lifestyle | 2 (3.7) |
|  | Supportive care | 2 (3.7) |
|  | Medical facility | 1 (1.9) |
| Primary outcome |  |  |
|  | Death | 35 (64.8) |
|  | Cancer incidence | 13 (24.1) |
|  | Patient well-being | 5 (9.3) |
|  | Pathological response | 1 (1.9) |
| Methods for outcome effects |  |  |
|  | Cox proportional hazards regression | 27 (50.0) |
|  | Pooled logistic regression | 16 (29.6) |
|  | Logistic regression | 8 (14.8) |
|  | Not mentioned | 3 (5.6) |
| Causal contrasts |  |  |
|  | PP | 38 (70.4) |
|  | ITT | 12 (22.2) |
|  | Both ITT and PP | 4 (7.4) |
| Randomization assignment |  |  |
|  | Clone | 14 (25.9) |
|  | PSM | 14 (25.9) |
|  | IPW | 10 (18.5) |
|  | ^b^Other | 8 (14.8) |
|  | PSM\IPW | 7 (13.0) |
|  | PSM\OW | 1 (1.9) |
| Selection bias |  |  |
|  | Clone-censor-weight | 14 (25.9） |
|  | PSM | 14 (25.9) |
|  | IPW | 10 (18.5) |
|  | PSM\IPW | 7 (13.0) |
|  | Not mentioned | 4 (7.4) |
|  | Logistic regression analysis | 3 (5.6) |
|  | Cox proportional hazards regression | 1 (1.9) |
|  | PSM\OW | 1 (1.9) |
| Time-varying confounding bias |  |  |
|  | Not mentioned | 31 (57.4) |
|  | Clone-censor-weight | 13 (24.1) |
|  | IPW | 6 (11.1) |
|  | G formula | 4 (7.4) |

"a" refers to the studies that directly compared treatment strategies involving different intervention models, for example, comparing the efficacy of surgery versus radiotherapy in the same population.

"b" refers to studies that used methods other than cloning, PSM, IPW, or OW to adjust for baseline confounders.

Abbreviations: ITT = Intention to treat; PP = Per-protocol; PSM = Propensity Score Matching; IPW = Inverse Probability Weighting; OW = Overlap weighting.
