# Supplementary material for: Target trial emulation studies in oncology: a comprehensive analysis
Source: Oncologist. 2026 Jul 7;31(11):oyag258. doi: 10.1093/oncolo/oyag258 (PMC13645428; doi:10.1093/oncolo/oyag258)
Supplement: Supplementary file 1 [file oyag258_supplementary_data.zip › Supplementary Figure.docx]

**Supplementary Figure 1**. Summary of cancer types in the 54 included studies, 2015-2023.

**Supplementary Figure 1**. Summary of cancer types in the 54 included studies, 2015-2023.

* "Multiple" refers to published literature exploring the TTE approach related to various types of cancer.

Abbreviations: RRMM = Relapsed or Refractory Multiple Myeloma; RRFL = Relapsed/refractory follicular lymphoma; ICC = Intrahepatic Cholangiocarcinoma; OGA = Oesophagogastric adenocarcinoma.
