# Supplementary material for: Target trial emulation studies in oncology: a comprehensive analysis
Source: Oncologist. 2026 Jul 7;31(11):oyag258. doi: 10.1093/oncolo/oyag258 (PMC13645428; doi:10.1093/oncolo/oyag258)
Supplement: Supplementary file 1 [file oyag258_supplementary_data.zip › Supplementary Material 1.docx]

**Supplementary Material 1**. Search Strategy

Supplementary Material 1. Search Strategy

Pubmed(

target trial emulation [Title/Abstract] OR

target trial [Title/Abstract] OR target trials [Title/Abstract] OR

index trial [Title/Abstract] OR index trials [Title/Abstract] OR

hypothetical trial [Title/Abstract] OR hypothetical trials [Title/Abstract] OR

hypothetical clinical trial [Title/Abstract] OR hypothetical clinical trials [Title/Abstract] OR

hypothetical pragmatic trial [Title/Abstract] OR hypothetical pragmatic trials [Title/Abstract] OR

hypothetical randomised trial [Title/Abstract] OR hypothetical randomised trials [Title/Abstract] OR

hypothetical randomized trial [Title/Abstract] OR hypothetical randomized trials [Title/Abstract]

)

and

(

neoplasms [MeSH Terms]

or

(

cancer* [Title/Abstract] or tumor* [Title/Abstract]

or tumour* [Title/Abstract] or neoplas* [Title/Abstract]

or malignan* [Title/Abstract] or carcinoma* [Title/Abstract]

or adenocarcinoma* [Title/Abstract] or choriocarcinoma* [Title/Abstract]

or leukemia* [Title/Abstract] or leukaemia* [Title/Abstract]

or metastat* [Title/Abstract] or sarcoma* [Title/Abstract]

or teratoma* [Title/Abstract]

)

)

Embase

(

'neoplasm'/exp

or

(cancer* or tumor* or tumour* or neoplas* or malignan* or carcinoma* or adenocarcinoma* or choriocarcinoma* or leukemia* or

leukaemia* or metastat* or sarcoma* or teratoma*):ti,ab

)

and

(

'target trial emulation':ti,ab OR

'target trial':ti,ab OR 'target trials':ti,ab OR

'index trial':ti,ab OR 'index trials':ti,ab OR

'hypothetical trial':ti,ab OR 'hypothetical trials':ti,ab OR

'hypothetical clinical trial':ti,ab OR 'hypothetical clinical trials':ti,ab OR

'hypothetical pragmatic trial':ti,ab OR 'hypothetical pragmatic trials':ti,ab OR

'hypothetical randomised trial':ti,ab OR 'hypothetical randomised trials':ti,ab OR

'hypothetical randomized trial':ti,ab OR 'hypothetical randomized trials':ti,ab

)

Web of science Core Collection Database

(

TS = (neoplasm

OR

cancer* OR tumOR* OR tumour* OR neoplas* OR malignan* OR carcinoma* OR adenocarcinoma* OR choriocarcinoma* OR leukemia* OR

leukaemia* OR metastat* OR sarcoma* OR teratoma*)

)

AND

( TS = (

"target trial emulation" OR

"target trial" OR "target trials" OR

"index trial" OR "index trials" OR

"hypothetical trial" OR "hypothetical trials" OR

"hypothetical clinical trial" OR "hypothetical clinical trials" OR

"hypothetical pragmatic trial" OR "hypothetical pragmatic trials" OR

"hypothetical randomised trial" OR "hypothetical randomised trials" OR

"hypothetical randomized trial" OR "hypothetical randomized trials"

)

)
